# American Association of Obstetricians and Gynecologists

**Published:** 1896-11

**Authors:** 


### Society Proceedings.

### AMERICAN ASSOCIATION OF OBSTETRICIANS AND GYNECOLOGISTS.

*Ninth Annual Meeting, held in Richmond, Va., September 22, 23 and 24, 1896.*

THE association met at the Jefferson hotel, and was called to order, at 10 A. M., by the president, Dr. Joseph Price, of Philadelphia.

THE CAUSE OF PELVIC DISEASE.

Dr. John M. Duff, of Pittsburg, read the first paper, which was entitled Pelvic diseases and their principal causes: what should the laity be taught concerning them? He said that notwithstanding the fact that some of the prominent members of the medical profession had, in talks to the galleries, held the gynecologists up to ridicule and criticised them severely, he did not think any apology was due either the profession or the public for the character or results of the work of pelvic surgeons. Those members of the profession who had been devoting themselves to the care of diseases peculiar to women, had, in the face of revilings and professional and public prejudice, worked patiently and persistently, until they were now obtaining results of which they may well feel proud, results far beyond what the most sanguine expectations of the hardy pioneers of a quarter of a century ago led them to hope for. They were today charged with irrational radicalism, with an operative mania, which was gratified without a proper consideration of the ultimate benefit to the patient. Entreatingly they were urged to adopt more conservative measures, and thus stop the wholesale mutilation which was going on at present, which it is claimed is neither scientific nor humane. Sentiments such as these, endorsed by men of reputation, were eagerly taken up by the lay press as sensational news, and advertised by pretenders as an endorsement of their methods of practice; and thus the laity, in the opinion of Dr. Duff, is taught false notions regarding the nature of pelvic diseases and their treatment. That there is a great amount of mutilation connected with pelvic surgery, he would not deny; but that regular pelvic surgeons were guilty of reckless despoliation was not, he thought, susceptible of proof. Pelvic surgeons could scarcely be held accountable for the work of general practitioners; and for the work of ignorant egotists and pretenders, who with brazen effrontery undertake operations for the performance of which they are not qualified by character, experience or education, the pelvic surgeons disclaim all responsibility. During the period of the evolution and upbuilding of pelvic surgery, no doubt much of the work was crude, and perhaps too much was done by over-zealous operators. That at this day, through mistaken diagnosis, operations are sometimes needlessly performed no one would have the hardihood to deny; but that such cases are as frequent as some critics say they are, Dr. Duff could not believe. He said the true pelvic surgeon was governed by nobler purposes, by more elevated aims. Conservatism in its true sense—the saving of life, relief from pain, the curing of his patient,—was his watchword.

### DECEPTIVE SIMILARITY OF SIGNS AND SYMPTOMS OF INTRAÄBDOMINAL DISEASE, WITH CASES.

Dr. Walter B. Dorsett, of St. Louis, followed with a paper on this subject. In order to arrive at a conclusion and to formulate a diagnosis in a given case, be it medical or surgical, the practitioner must exercise care and judgment in the consideration of such signs and symptoms as are presented. Each should be weighed and mental annotations taken as to their value individually and collectively. Dr. Dorsett directed attention to the importance of the family and personal history and habits of patients, to the pulse and temperature, the knowledge to be gained by manual examination, the use of analgesics, and the like. Regarding the exploratory incision, it should not be regarded as an evidence of ignorance, but as a legitimate means of diagnosis, and the off-hand diagnostician or the surgeon who never makes mistakes should be looked upon with at least a grain of suspicion. To illustrate his statements he reported the following case :

Mrs. M., aged 28, married eight years, no pregnancies, was seen by Dr. Dorsett about a week after having recovered from an attack of malarial fever. Temperature, 99° F.; pulse, 90 ; tongue slightly coated, and a tendency toward diarrhea. Complained of general abdominal tenderness. Palpation of abdomen revealed a slightly more tender spot at McBurney's point ; no swelling or tumefaction could be felt. A vaginal examination revealed a retroversion with fixation ; no tubal enlargement nor tenderness could be made out, and no vaginal discharge. Diagnosis : gastro-intestinal irritation, with chronic inflammation of pelvic contents. Diarrheal mixture was prescribed and patient was told that further attendance would probably not be necessary. Four days subsequently the temperature was 99.8° F.; pulse, 100. Abdominal palpation revealed a distinctly tender spot with some swelling at McBurney's point. Patient stated that she had eaten heartily of wienerwurst the day before and had been awakened during the night by cramps at the navel. Bimanual examination was again resorted to, with negative results. Appendicitis was diagnosticated at this visit, first stage. Drachm doses of salts were prescribed and patient was urged to go to the hospital, but refused. The next day she was found sitting in a rocking-chair, and aside from slight tenderness over abdomen was feeling quite comfortable. The salts had acted freely. Bimanual examination again gave negative results. Temperature,

99° F.; pulse, 100. Patient was ordered to bed and advised to keep quiet. At that time she was regarded as better, and thought to be out of danger, but the following day the pain became more severe, and the patient came to the hospital of her own accord. Upon examination the right iliac fossa was found to be exceedingly tender and fluctuating. Vaginal examination revealed nothing aside from what was found at the previous examination. Temperature, 103° F.; pulse, 130. Diagnosis: ruptured appendiceal abscess. She was anesthetised and placed upon the table, and a section made in the median line. The large sac was found on the right side filled with fluid blood and clots, and when washed out a rent of the posterior layer of the broad ligament was found, which communicated with another rent in the Fallopian tube. The appendix was perfectly healthy and was not disturbed. A thorough washing out of the sac was done, and ligation of the tube with a portion of the broad ligament; a glass drainage-tube was introduced. Notwithstanding the utmost care, the temperature remained high, the pulse became worse, the abdomen became distended, and the patient died on the third day. Post operative diagnosis: ruptured tubal pregnancy, without the usual symptoms. There were no history of shock, no cessation of menstruation nor any nervous symptoms of pregnancy, no passage of decidua, no vaginal discharge of any kind; but in their stead a good history and train of signs and symptoms of inflammatory disease of the appendix.

### THE MOST POTENT CAUSES OF PELVIC INFLAMMATION.

Dr. Rufus B. Hall, of Cincinnati, read this paper. He claimed that septic infection following labor or abortion, or gonorrheal infection, was the cause in almost every instance. He said there would always be some cases of septic infection following labor, which are in no wise due to infection from the attendant injury to small pelvic tumors, and the like. The retention of the products of conception in abortion is a very frequent cause. He advised complete emptying of the uterus at once after abortion. He believed the most frequent cause to be gonorrheal infection conveyed to the woman from a latent gonorrhea of her husband. The more he saw of the ravages of gonorrhea, the more he was convinced of the fact that physicians are derelict in their duty to their patients in the dissemination of knowledge upon this subject. The teaching of a few years ago that gonorrhea in the male could be easily and speedily cured by a little balsam of copaiba or oil of sandal wood, with mild astringent injections, and that the patient was well as soon as the purulent discharge ceased, is false doctrine and must be corrected. This must be done by the family physician. Dr. Hall said that on many occasions he had been compelled to remove suppurating tubes and ovaries from women who had contracted the disease from husbands who believed themselves well when married. He had no hesitation in saying that gonorrhea is more destructive to women than syphilis, and believed it is the duty of every physician to impress upon the male patient the fact that he is not well as soon as the urethral discharge disappears. He was a firm advocate of legislation upon this subject, believing that every man should have a certificate from the health officer of freedom from syphilis and gonorrhea before he could obtain a marriage license.

Dr. J. HENRY CARSTENS, of Detroit, in discussing Dr. Dorsett's paper, said that the difficulty attending diagnosis in some cases was exceedingly great. The gynecologist should exhaust his diagnostic resources before resorting to abdominal section. The too frequent opening of the abdomen stimulated incompetents to do likewise, and as a consequence results were disastrous, eventually reacting on gynecologists.

Dr. W. E. B. DAVIS, of Birmingham, Ala., did not believe that gonorrhea played so important a part in the production of pelvic inflammation as had been asserted. One's conception of causes of pelvic trouble depends largely upon the class of patients one has. The cases met with in dispensary practice are different from those encountered in private practice. He believed that fully 50 per cent. of the cases of pelvic inflammation are due to puerperal infection, either at the time of delivery at term or of premature delivery. As to tuberculous trouble, more importance is being attached to it as a cause of pelvic inflammation than it deserves. Those who did considerable operative work knew that only a small percentage of cases have their origin in tuberculosis.

Dr. JAMES MCFADDEN GASTON, of Atlanta, called attention to the prophylactic management of cases of pregnancy prior to the period of confinement. Extreme hygienic precautions might warrant in some instances the use of antiseptic douches prior to labor, but there was a great tendency on the part of some members of the profession to resort to measures which are regarded as precautionary, and to order douches in advance of confinement. He believed that this was altogether out of place, for when there is a normal condition of things nature should be allowed to take its course.

Dr. ERNEST S. LEWIS, of New Orleans, cited a case illustrating the errors that sometimes arise in the diagnosis of abdominal tumors. He operated on a patient last winter for what he supposed at the time was a small ovarian tumor, but after the abdomen was opened it turned out to be a retroverted gravid uterus.

### TUBO-OVARIAN CYSTS.

Dr. Albert Goldspohn, of Chicago, read a paper on this subject. By tubo-ovarian cyst is meant a nonpurulent sac whose walls are composed in variable proportion, of the walls of the Fallopian tube and those of some cystic ovarian or parovarian formation, with the coalescence of two or more cavities—at least one from each—into one, by a free communication. The fluid contents of such a sac may be serous or hemorrhagic, or may partake, in variable degree, of the qualities and characteristics of the fluid contained in glandular ovarian cystomas. The fimbriæ of the abdominal ostium of the tube may be distinguished or not upon the inner or on the outer side of the ovarian portion of the sac, or they may have coalesced with other structures to form some portion of the walls of the united sac. The ovarian element in this formation can have originated from a hydropic Graafian follicle, a cystic corpus luteum, from the primordial glandular ducts of Pflueger in the ovary, or from the parovarian. In order to exclude a large number of ordinary tubo-ovarian conglomerates, we need to recognise the following minimum requirements in distinguishing a tubo-ovarian cyst: (1) The participation of the tube, which is easy enough from its position and connections. (2) To prove the participation of the ovary by demonstrating some ovarian tissues in the wall of the sac. (3) That their cavities are united by some opening through which the mucous membrane of the tube is continuous with the lining of the ovarian cyst or follicle. The following were the conclusions of the paper: (1) Tubo-ovarian cysts come to pass in consequence of a plastic inflammatory union between a Fallopian tube and the adjacent ovary, after either or both of these organs and the intervening peritoneum have experienced a nonpurulent pathological change of a cystic character, the septum intervening between the two lumina disappearing in consequence of pressure atrophy from the tension of liquid confined to one or both sides of it. (2) This union of a distended tube cavity may occur also with that of a parovarian cyst (v. Ott) or with that of a peritoneal pseudo-cyst (Zedel). (3) In those rare cases in which the fimbriæ are really found floating in the interior of the main cyst cavity, we must assume either the congenital anomaly of an "ovarian tube,"

as was seen by Schneidemahl in a mare, as a *vitium primæ formationis*, or that an ovarian cyst or follicle cyst ruptured, and the abdominal end of the tube dropped into the rent and was united to its edges by inflammatory action, thus making a joint cyst and tubal cavity.

MIXED TUMORS OF THE OVARY.

Dr. Walter B. Chase, of Brooklyn, followed with a paper with this title. Mixed tumors of the ovary have a peculiar interest, for the reason that, if small, they are often difficult of diagnosis. These tumors of the ovary may be made up of a variety of cysts, or may be a combination of cysts and solid growths. The etiology of tumors, as a whole, is a matter of great importance, both in the relation to diagnosis and treatment. The question of what constitutes a tumor might be considered with profit. Senn defines a tumor as "a localised increase of tissue proliferation of embryonic cells of congenital or post natal origin." An important fact concerning true tumors is that they never disappear except by removal or destruction. Benign tumors always remain local, while malignant ones are disseminated by migration or transportation of their peculiar cells, and they always originate as benign or malignant growths. If the tumor matrix is made up of embryonic cells of the lowest development, there is greater liability to malignant growth than if from tissues susceptible to the highest physiological type of development. Retention cysts of the ovary are not tumors in a technical sense, and they never attain large size. Large ovarian cysts are most often cystadenomas and are not developed from Graafian follicles, but arise from the embryonic structure. It would seem, then, that the genesis of simple and mixed tumors is divested of much that is misleading and contradictory and reduced to a rational basis. It also demonstrates with great clearness that tumors are not only of local origin, but at their inception are congenital.

MOVABLE KIDNEY: LOCAL AND REMOTE RESULTS.

Dr. A. H. Cordier, of Kansas City, Mo., read a paper on this subject, in which he drew the following deductions: (1) A movable kidney often produces a dilatation of the stomach with all the accompanying symptoms of a disease of that organ. (2) It is a fruitful source of gall stones, because of the pedicle producing a partial obstruction of the common duct. (3) The bending of the ureter often gives rise to a hydronephrosis. This, in turn, is sometimes converted into a pyonephrosis. (4) It may produce death by a complete strangulation by a torsion of the vessels and ureter. (5) By dragging on the abdominal aorta and kinking the vena cava, a condition simulating an aneurism of these vessels may be produced. (6) Pain of a referred character to the region distribution of the spinal nerves is often induced by a movable kidney's disturbance of the abdominal brain. (7) A general nerve exhaustion (neurasthenia) is often induced by the interference of this condition with digestion, assimilation and elimination. (8) Nephrorrhaphy is a safe and effective surgical procedure. (9) All cases of movable kidney, if accompanied by symptoms pointing to the kidney as their source, should be operated on. (10) In summing up the local and remote results of this now often recognised condition, the author thinks the correctness of the deductions has been frequently demonstrated by the disappearance of each and every symptom after a restoration and retention of the kidney in its normal position. (11) Symptoms are not to be relied upon in making a diagnosis of movable kidney. The physical examination is the only trustworthy guide.

THE LIMITS OF NEPHRORRHAPHY

Was the subject of a paper by Dr. HUGH M. TAYLOR, of Richmond, Va. He conceded the frequency of nephroptosis. Since he had been sytematically looking for movable kidney, he had found it so frequent in its occurrence that he no longer regarded the experience of Glénard, Lindner, Edebohl and Noble as unique. His opinion was equally fixed that only a small proportion of the cases met with give rise to symptoms of suffering, ill-health or death, and consequently a majority of cases do not call for nephrorrhaphy. He favored the classification of nephroptosis under three clinical heads : (1) Patients who have displaced kidney do not know it, and suffer no inconvenience whatever from it. This type he thinks represents by far the largest class. (2) Patients with displaced kidney, who may or may not know it, who suffer from gastro-enteric discomfort and perhaps a long train of vague neurotic disturbances. In this type he thinks we find the largest class calling for operative interference. (3) Patients with movable kidney who are subjects of occasional or frequent mild or severe attacks of renal crises. This last mentioned is, he thinks, the least frequent type met with, but the urgency of the symptoms more frequently demands operative interference. Nephrorrhaphy for the relief of gastro-enteric disorder is limited by our ability to tell to what extent the disorder is due to renal ptosis *per se* or to enteroptosis, or to some one of the many well-known etiological factors of gastroenteric disorder. Nephrorrhaphy for the relief of the condition of Deitl's or renal crises must be limited by one's success in differentiating between this condition and that of gall-tract, appendicular and kidney colic due to nephrolithiasis. He accepted as logically sustained the conclusion that the Deitl's or renal crisis is due to a kink or twist of the ureter with retained urine in the ureter and pelvis of the kidney. Apart from the violent paroxysms of pain (the renal crisis), the tendency of ureteral twist and urinary obstruction to induce hydronephrosis and in exceptional instances pyonephrosis rendered operative interference more imperative in this class of cases. His protest was not against nephrorrhaphy, but against its abuse. He conceded the value of operative interference in many selected cases, but deprecated the tendency toward operative interference merely because the kidney is movable.

Dr. George Ben Johnston, of Richmond, Va., said that some years ago his attention was called to the subject by encountering several cases of movable kidney that had been unobserved either by him or by the physician who preceded him in the treatment of these cases for obscure nervous and gastro-intestinal disturbances, and when he observed the similarity of symptoms in the first three cases which he saw he was obliged to associate those symptoms with the presence of movable kidney. He prevailed on these women to be operated upon for movable kidney and in all three cases the results were most gratifying.

Dr. L. H. Dunning, of Indianapolis, was greatly interested in the subject, for the reason that about 1880 he resorted to operative procedures for the cure of floating kidney, and in connection with this work he sought to determine, if possible, some of the causes which led to movable kidney. He emphasised the importance of differentiating between floating and movable kidney, the former being always congenital, the latter acquired to a greater or less extent. He found by his investigations that the partially fixed condition of the kidney depended upon three or four causes, the two principal ones of which were its position behind the peritoneum and the fact that it had an envelope of cellulo-adipose tissue. A little further investigation showed that the perinephritic cellulo-adipose tissue was composed of two parts, one fixed, the other movable. The normal kidney had a range of motion of from one-half to three-quarters of an inch in its fatty envelope.

Dr. THOMAS B. EASTMAN, of Indianapolis, reported the case of a woman, twenty-five years of age, who came to him with the symptoms of appendicitis. She also had considerable albumin in the urine. Operation showed that the appendix was firmly adherent to the kidney. It required considerable force to liberate it. As soon as liberated the kidney bounded back into place as though it were rubber. The appendix was removed, the albumin in the urine ceased, and the woman made an uneventful recovery.

Dr. JAMES MACFADDEN GASTON, of Atlanta, directed attention to the possibility of movable kidney being mistaken for enlarged gall bladder. The gall bladder was capable of being pushed back into the lumbar region and carried around in front in just the same manner as a floating kidney. It behooved gynecologists to look into this phase of the matter.

Dr. W. E. B. DAVIS, of Birmingham, had seen a number of cases of movable kidney, and said that at the Charleston meeting of the Southern Surgical and Gynecological Association there was quite a difference of opinion as to the frequency of the condition. He believed that movable kidney was a condition which did not require in all cases operative interference. Of the number of cases he had seen he had only operated on a few.

Dr. I. S. STONE, of Washington, D. C., related the case of a woman who, after the operation of nephrorrhaphy had been performed, gained twenty-five pounds in flesh. In many instances this procedure brought color back to the cheeks of patients and made them feel well. He had never seen such gratifying results from any other operation in surgery, except, perhaps, the removal of an ovarian tumor. The patient made rapid improvement after the operation.

Dr. JOSEPH PRICE, of Philadelphia, said his experience was somewhat limited in operating for movable kidney. The improvement in the condition of patients so operated upon was rapid, but there was such a thing as operating too much upon cases of movable kidney.

Dr. J. HENRY CARSTENS, of Detroit, said the line should be drawn between movable and floating kidney. The trouble which arose from floating kidney consisted of a twisting of the ureter and consequent obstruction.

TREATMENT OF PERIUTERINE SEPTIC DISEASES.

Dr. W. E. B. DAVIS, of Birmingham, Ala., read a paper on this subject. Only recently has the extremely radical procedure of hysterectomy been practised in this country for septic diseases of the internal genitals. A wave which had its origin in Paris at the hands of Péan, aided by Richelot, Segond, Jacobs and others, reached our shores three years ago and has found a considerable following among our leading operators. The claim is made that there is no use in leaving the uterus behind after the removal of the appendages. In every operation for septic diseases of the female generative organs which demands the removal of the tubes and ovaries, hysterectomy should also be performed, unless there are plain contraindications forbidding it. It should be the aim of the surgeon to preserve everything consistent with thorough surgical work, and not to sacrifice important organs because it can be done with only a small mortality. We are told that the uterus has no function after the removal of the appendages, but this has not been demonstrated; and, on the contrary, we know that the sexual life of the woman is very much better preserved by leaving the uterus, and that the mental effect is also much better. A slow convalescence, or even a second operation, is preferable to its removal, unless very much diseased. It is a reflection on the correctness of the reports by many most excellent surgeons of complete recoveries of such a large per cent. of the cases when the uterus was not removed, to accept the argument now being used in favor of hysterectomy in all these cases. As stated by Dr. Davis at the last meeting of the American Medical Association, he could not agree with Dr. Sutton and others that pus in the tubes was due to gonorrhea in 75 per cent. of cases. He thought that puerperal infection was the cause of more than 50 per cent. Tuberculous infection was rarely the cause, and was not so important as had been claimed. However, the importance attached to gonorrhea was against the argument for the removal of the uterus, as the infection from this source was not deep and could be removed with the curette. Because some patients were not completely cured by the removal of the appendages was no argument for hysterectomy in every case in which the bilateral operation was required; for nearly all these could be relieved by a thorough curettage. Some large uteri would require, in addition to this, a high amputation of the cervix, and only a small number would need a hysterectomy. Vaginal incision for the drainage of pus in the pelvis, not confined to the tubes, was a most valuable method of treatment in a well-recognised class of cases, and had been practised for a long time, with gratifying results. A large number of these cases required no further surgery. More recently large pus tubes and ovarian abscesses had been incised and drained through the vagina, with permanent recoveries in a good proportion of cases. The uterus should always be curetted at the same time. These were the very cases in which the vaginal operation and hysterectomy had been recommended so highly by the French surgeons. Yet a considerable percentage of these cases could be relieved by vaginal incision and drainage. The object of the surgeon should be, not so much to reduce still further the death-rate from the operation, but to relieve the subjects and preserve as far as possible organs which had so much to do with the woman's health and happiness.

HYSTERECTOMY IN THE PRESENCE OF ACTIVE INFLAMMATION.

Dr. L. H. Dunning, of Indianapolis, followed with a paper upon Hysterectomy in inflammatory diseases of the pelvic organs. The author discussed only that form of inflammation of the pelvic organs and tissues denominated diffuse pelvic inflammation, and drew the following conclusions:

1. We recognise the utility of hysterectomy in a small percentage of cases of bilateral suppuration of the tubes and ovaries, in which the uterus is distinctly septic, and in cases of septic uteri which cannot be cured by other means after bilateral salpingo-oöphorectomy.

2. We oppose hysterectomy, as a rule, in inflammatory diseases of the pelvic tissues upon the following grounds—namely, (*a*) The uterus is the central organ of the reproductive system, and should not, except upon palpable and urgent cause, be extirpated; (*b*) it is only in rare cases that the uterus is so far diseased as to resist the curative effects of appropriate treatment; (*c*) the removal of the uterus profoundly affects the nervous system and emotional nature of young women deprived of this organ; (*d*) we oppose the removal of the uterus from anatomical reasons—namely, as a result, the vagina is shortened; the anatomical relations of the bladder, sigmoid and rectum are changed; the elasticity of the pelvic diaphragm is greatly diminished or entirely removed, the elastic tissue being largely replaced by sensitive scar tissues; (*e*) in married women it often disturbs the sexual relations of husband and wife, and is apt to induce mental depression; (*f*) vaginal hysterectomy compels the use of drainage, because of the necrosis of tissue and suppuration induced.

SHALL THE UTERUS BE LEFT IN SITU IN EXCISION OF THE ADNEXA ?

Dr. E. F. Fish, of Milwaukee, Wis., read a paper which was an argument in favor of leaving the uterus in situ, if sound, after excision of the appendages. It considered the pathological conditions requiring hysterectomy after salpingo-oöphorectomy, as well as the conditions which do not require it. The author argued against all operations which leave a degenerated uterus, such as Hegar's, Tait's, Martin's and Robinson's, except under extreme conditions, and concluded as follows : 1. Whenever it becomes necessary to excise the uterine adnexa, if the uterus is sound, leave it. 2. Whenever we excise the tubes and ovaries, and the uterus, though in a pathological condition, in our judgment will yield to treatment, leave it. 3. Whenever it is necessary to do an abdominal hystero-salpingo-oöphorectomy and the cervix is healthy, do a supravaginal amputation, as this leaves the vaginal vault intact. 4. Whenever it is necessary to do a supravaginal amputation, suspend the cervix to the stumps of the broad ligaments or anchor it to the abdominal wall, to prevent prolapsus vaginæ. 5. Whenever it is necessary to do a general ablation, and the cervix uteri is unsound, take the entire organ, because of the danger of carcinoma. 6. Whenever a subserous or interstitial myoma can be removed without too great damage to the uterus, do a myomectomy and leave the organ. 7. Whenever we excise the appendages and leave the uterus, ventral fixation is not an unsurgical operative conclusion.

The author's reasons for leaving the uterus were that it helps to maintain the woman's sexual integrity ; it relieves the patient of much mental strain, and is a prophylactic measure to neurasthenia, melancholia and insanity ; it tends to maintain the family ties unstrained ; it obviates the possibility of vaginal hernia, cystocele and proctocele and delays vaginal atrophy ; and, finally, it holds up and prevents shortening of the vagina.

DYNAMIC ILEUS.

Dr. J. W. Long, of Richmond, Va., contributed a paper with this title. Intestinal obstruction had been variously classified, but Dr. Long regarded the classification adopted by Murphy as the simplest and the most rational : 1. Adynamic ileus, always the result of intestinal paralysis, due to varying causes, may be clearly illustrated by such cases as those following injury to the spinal cord and paralysis due to peritonitis. 2. Dynamic ileus. This variety formed the subject of the paper and was discussed in detail. 3. Mechanical ileus embraced such common lesions as strangulated hernia, intussusception, fecal impaction and the like. The speaker reported the following case :

Mrs. C., 21 years old, married three years, but never pregnant ; was rather below the medium size and height. In temperament she was of the spoiled child type, not hysterical but rebellious. It was with great difficulty that she could be induced to have any local treatment or even to take medicines. After admission to hospital her obstreperous disposition required all the tact and firmness of a sagacious nurse. Early in April of this year the patient had malaria, followed by delayed menstruation, pelvo-abdominal pain and obstinate constipation. The malaria and menstrual disturbance yielded promptly to treatment, but the abdominal pain continued, and gradually the ileus symptoms became more and more pronounced. After exhausting every other measure to move the bowels, the patient was put under the influence of chloroform and by means of a Ricketts tube a quantity of fecal matter was washed away. Notwithstanding there was no improvement, the nausea and vomiting recurred oftener and were most distressing, the pain and tenderness became worse, and a marked degree of tympany supervened. When she was brought to the hospital there had been no movement of the bowels for four weeks, excepting what was washed away with the colon tube while the patient was anesthetised. The history justified the diagnosis of intestinal obstruction, while the urgent symptoms demanded an immediate operation. The abdomen was opened by a median incision. No mechanical obstruction could be found, although a careful search was made along the whole length of the intestine. The bowel was moderately distended with gas and congested. A singular feature, however, was that at three points—two in the ileum and one in the sigmoid flexure—the canal was constricted sufficiently to constitute obstruction. In the ileum one of the constrictions was about fifteen inches from its lower end and six inches long ; the other was nearer the jejunum and about four inches long. The lumen was not entirely closed at either point, but was greatly reduced, being less than half the normal size ; while the diameter of the remaining portions of the bowel was increased, on account of the distention with gas. No peristalsis was observed, but the contracted portions could be dilated by "milking" the intestinal contents along. In the sigmoid the limitations of the contracted portion were not so sharply defined, but the lesion was just as evident. The walls were thickened and the caliber much diminished. Incidentally a small ovarian cyst on the right side was discovered and removed. As the intestine had been handled a good deal, the abdomen was flushed with normal salt solution. The incision was closed with two tiers of sutures—silk for the peritoneum and interrupted silver wire for the remaining layers. The recovery was most satisfactory in every way. The bowels responded to the usual laxatives and enemas on the second day, and from the first to last there was not a hitch in her convalescence. The patient left the hospital in four weeks, and three weeks thereafter took a trip to Alabama. There could be discovered no evidence of lead or ptomain poisoning.

### SPONTANEOUS RUPTURE OF THE UTERUS DURING LABOR AT TERM.

Dr. B. M. Hypes, of St. Louis, read a paper on this subject :

Mrs. O., aged 31, of German parentage, general health good ; had had one child four years ago. Labor pains began September 16, 1895, at 10 P. M., at full term. The family physician was called, and found labor in progress, vertex presentation, with normal condition of mother and child. The pains were slight and progress was slow. At 2 A. M., September 17th, he gave a dose of morphine and went home. At 9 A. M., upon his return, he found the patient comfortable, with occasional slight labor pains. He left the house, with injunctions to call him when signs of labor became pronounced. Patient remained quiet during the day. Suddenly, at 3 P. M., she was seized with violent vomiting, followed by the most excruciating pains in her abdomen, associated with rolling and tossing in bed, gasping for breath, faint feelings, pallid face and rapid exhaustion ; in short, the usual symptoms of abdominal shock. The family physician was at once sent for, and upon his arrival, at 4 P. M., found her in complete collapse, with convulsive seizures. The symptoms, with vaginal and abdominal examination, revealed to him this dreadful condition : the presenting part receded, the womb empty and the child plainly felt in the abdominal cavity. The patient had suffered spontaneous rupture of the uterus. The physician at once sent for surgical aid ; but by the time the surgeon, Dr. Meisenbach, arrived, the patient was moribund. Still, with the hope of saving the child, laparatomy was hastily performed ; and the child, which had escaped entirely into the abdominal cavity, was extracted from a mass of blood and amniotic fluid. It had ceased to live, and continued efforts at resuscitation failed to cause it to breathe. The child was fully developed, male, weighed six pounds and was eighteen inches long. The uterus, when removed from the body, presented the following condition: a rupture through the fundus superiorly, extending from half an inch from the entrance of one tube to an equal distance from the entrance of the other ; the walls at the place of rupture were comparatively thin. The placenta was located at the middle third of the uterus, anteriorly and to the right, where the walls were much thickened. The vaginal portiǒn of the cervix was almost obliterated, as at term, and dilated for the ready admission of two fingers. The lower zone of the uterus exhibited no thinning or formation of Bandl's contraction ring ; there was no disease of tubes, ovaries or placenta. A microscopical examination was made soon after rupture, and revealed fatty degeneration of tissue at the point of rupture.

PORRO'S OPERATION.

Dr. EDWIN RICKETTS, of Cincinnati, O., reported a case of Porro's operation at or near the fifth month for small fibroid of cervix, accompanied by hydramnios and total retention of urine :

Mrs. M., white, aged 26, of short stature ; the mother of two children, of six and three years of age ; had had an abortion at eight weeks early in 1895 ; there was no specific history. She was a patient of Drs. J. B. and C. M. Warwick, of Lucasville, O. On February 23, 1896, she had severe labor pains, lasting thirty-six hours and accompanied by slight hemorrhage. The right portion of the cervix was soft and the left hard, which condition was also present at the time of operation. During April and until May 22d, the date of operation, she had great tenderness over the lower part of the abdomen, and at times had a temperature above 100° F., with a pulse running from 90 to 100. Dr. Ricketts saw her in consultation at her home, April 8, 1896, when for the first time motion of the fetus was barely perceptible. On May 22d, Drs. Warwick, Kline, Sellards and Ricketts found her abdomen larger than it should be at full term, which was due to the hydramnios present. There was no difficulty in moving the fetus freely in the abdominal cavity, so thin was the uterine wall. It was considered unwise to delay surgical interference, and a Porro operation was therefore performed, under as strict asepsis as the circumstances would permit.

After the abdomen was opened, Dr. Ricketts passed his hand down into the pelvis, breaking up the pelvic adhesions. Upon the delivery of the fundus of the impregnated uterus through the abdominal incision, a rubber ligature was thrown around it, low down and tight enough to control any hemorrhage which might occur. The fluid which escaped, upon opening the uterus, surpassed in amount any he had seen delivered *per viam naturalis*. After carefully sponging the parts, the wire was tightly adjusted below the rubber ligature by means of the Këberlé clamp, and the rubber ligature then removed. After the delivery of the placenta, the fundus was amputated, leaving the ovaries and tubes intact. The abdominal wound was closed with silkworm-gut sutures, without stitching any tissue to the stump below the wire. No drainage-tube was used. The extraperitoneal part of the stump was dressed with gauze, moistened in glycerin and tincture of iron, the stump being held up by the double-hooded pin of Tait. The placenta and fetus were small for one of nearly five months' gestation, and the cord was tied in almost a hard knot—harder than any he had seen. The fetus had marked cyanosis and gasped but once. Recovery of the mother was satisfactory.

### TREATMENT OF PUERPERAL INFECTION.

Dr. H. W. Longyear, of Detroit, first spoke of the prophylaxis and, under this head, of the difficulty of securing reliable statistics regarding puerperal mortality of patients under the care of midwives in this country. The prophylaxis was divided into general and specific. He spoke of the treatment of infection from abortion and from childbirth at full term, and presented an instrument designed by him for use in removing the remains of secundines from the uterus. He also exhibited a self-retaining drainage-tube of his own invention and demonstrated its applicability. He reported two cases of puerperal infection treated successfully by the use of diphtheria antitoxin serum. He condemned the performing of hysterectomy for puerperal septicemia, except in very exceptional cases.

### ATRESIA WITH RETENTION OF THE MENSES—TREATMENT.

Dr. William H. Meyers, of Fort Wayne, Ind., read a paper with this title. The author reported two cases of atresia, one with absence of the vagina and uterus, and the other with retained menstrual fluid. The last was operated upon successfully. He believes that in a case of atresia of the vagina with retention of menstrual fluid in the uterus, an operation ought to be completed at one sitting, the direct method being adopted. He thinks the teaching in a recent work, that "the best way is to make a small opening into the mass and allow the contents to flow away gradually," is not sound. He could not, therefore, see in rapid evacuation such great dangers as were referred to in the books.

### PRINCIPLES AND PROGRESS IN GYNECOLOGY.

Dr. Joseph Price, of Philadelphia, delivered the president's address. He first thanked the association for the distinguished honor conferred in electing him president, which he said was the most gratifying expression of personal and professional kindness. He said the association was made up of earnest, enthusiastic and eminent men of the medical profession. We had more than a passing interest in the record of the transactions of our medical and surgical associations. From them the history of the progress of medical and surgical science would be made up; they would reflect the advanced thought and opinions, the strength of the endeavors, the results of clinical experience and research of the profession of this period. We had the inspiration of the reflection that our high service was that of humanity, and, Dr. Price said, the members were there to learn through the interchange of the best counsel how to make that service the best.

### SOME CAUSES OF INSANITY IN WOMEN.

Dr. George H. Rohe, of Sykesville, Md., read this paper, of which the following is an abstract: The general causes of insanity are the same in women as in men, but there are modifying conditions in the life history of men and women that influence the causation of mental disturbance as between the two sexes. General paresis and alcoholic insanity are more frequent in men because the latter are exposed to their causes to a greater degree and intensity. Menstrual, puerperal and climacteric insanity are, on the other hand, self-evidently limited to women. Women are especially subject to mental disturbances, dependent upon their sexual nature at three different periods of life: puberty, the child-bearing period and the menopause. The functions and activities peculiar to these periods have an intimate etiological relation to certain insanities. It is probable, however, that these functions have no influence in the production of insanity in their normal condition. It is only when the functions are disturbed or when pathological conditions are present that they have any unfavorable influence upon the psychical functions. At the period of puberty, menstrual derangements are not infrequently causative of mental disturbances which do not yield until the menstruation becomes normal. In the puerperium, insanity is dependent upon septic absorption or upon the consequences of other morbid conditions of the reproductive organs. Lactational insanity may be due to physical exhaustion, but in some cases pathological conditions of the genitals or of the breasts seem to have an etiological relation. At the menopause the disturbances of nutrition associated with the arrest of menstruation often produce insanity, and in many of these cases there will also be found abnormal alterations of the reproductive organs. The insanities following gynecological operations are either due to septic conditions or are merely due to the rapidly induced menopause. Their frequency has been much exaggerated.

### THE RELATIONS OF VISCERAL DISORDERS TO THE DELUSIONS OF THE INSANE.

Dr. Walter P. Manton, of Detroit, Mich., said that the delusions of the insane are often an expression of somatic peripheral irritation has long been recognised, but observation leads Dr. Manton to believe that the importance of these mental manifestations as indices of bodily suffering was frequently ignored and they were regarded as a mere phase of the brain disorder, especially in the instance of supposed fancied visceral disturbances. For convenience of consideration, he placed the so-called visceral lesions in four classes: (1) Delusions arising *de novo* from the diseased activity of the brain. (2) Delusions regarding external or visible abnormal bodily conditions. (3) Delusions arising from easily determined visceral disorders. (4) Delusions dependent upon obscure abdominal and pelvic states.

### OÖPHORECTOMY FOR THE INSANITY AND EPILEPSY OF THE FEMALE.

Dr. David T. Gilliam, of Columbus, O., contended in this paper that oöphorectomy was a logical and legitimate operation for the epilepsy and insanity of the female. Insanity is heredity, as is also epilepsy. They constitute the greatest curse to humanity. An insane father or an insane mother brings more misery into the world than any other father or mother. The offspring of such a parent, when ushered into the world, would be confronted by the awful spectacle of impending doom, and though he called on the rocks or the mountains to fall on him, the curse would pursue and overtake him. Dr. Gilliam then gave a picture from real life. He would limit the operation to those in whom the malady appears in some way to be connected with or dependent upon sexual disturbance. He would go further and include all who were willing to undergo the operation to save themselves and their offspring from the miseries which awaited them.

### TREATMENT OF THE STUMP TO PREVENT ADHESIONS.

Dr. J. F. Baldwin, of Columbus, O., followed with a paper on this subject. He estimated that about 1 per cent. of all subjects operated upon die from intestinal obstruction, the result of adhesions to the stump. To diminish as much as possible the danger of adhesions, he recommended the careful closing in of stumps by a peritoneal flap, and described the method of securing this flap. In cases in which the pedicle is, after a simple ovariotomy, not too large, he recommended that the pedicle be so ligated that the ends of the ligature were on the anterior face of the pedicle; that the ends of the ligature be then carried across the face of the stump, down and through the broad ligament, transfixing the ligament from behind forward. The ligatures should be passed through about half an inch apart. As the ends were drawn through and tightened, the raw end of the stump should be rolled down and under the broad ligament, so as to be entirely protected. He had used this method in a large number of cases and with entirely satisfactory results.

ABDOMINAL SECTION FOR TUBERCULOUS DISEASE.

Dr. THOMAS E. McARDLE, of Washington, D. C., reviewed briefly what has already been done by surgical means for the relief of women suffering from tuberculosis of the generative organs. There is no doubt that tuberculous disease of the female genitalia is more frequent than is generally supposed. Every portion of the genital tract may be affected, the order of frequency for the various portions being the tubes, body of the uterus, ovaries, vagina, cervix and vulva. The tubes are affected in nearly all cases, the body of the uterus in about three-fourths of the cases and the ovaries in about one-half of all cases. Tuberculosis of the body of the uterus is not at all a rare affection, and has been frequently discovered in autopsies upon phthisical subjects. It can be the only focus of disease in the body, but it is generally associated with disease of the tubes and is secondary to disease of that organ. Of all the female genitalia, the vulva is the least liable to tuberculous infection.

MELANO-SARCOMA OF THE FEMALE URETHRA.

Dr. CHARLES A. L. REED, of Cincinnati, O., reported this case:

Mary E. Y., aged 64, single; brought to his private hospital Decem-3, 1895. The patient had had no previous serious illness. There was no history of tuberculosis or syphilis in the family. The virginal condition of the genitalia precluded the supposition of venereal infection of any character. Her general health was good, although there was some emaciation about the neck and breasts, the latter of which were flabby—changes no doubt incident to age. Careful examination revealed no diseased conditions about either the lungs or the heart. Careful palpation and percussion of the abdomen yielded negative results. About eight months previously,—*i. e.*, in April, 1895,—she began to notice some pain, accompanied with blood, on micturition. This was shortly followed by a more or less pinkish discharge from the genital fissure. The self-examination which followed revealed a tumor at the meatus urethræ. This tumor continued to increase in both size and hemorrhagic tendency until she was prompted to consult Dr. Morris, who curetted the neoplasm thoroughly and treated it with styptics. When the patient came under Dr. Reed's care he found a black lobulated and eroded mass, about three centimeters in diameter, separating the labia majora. The orifice of the urethra was in the very center of this mass. A careful vaginal examination was not made at the time, as the virginal structures, present in their integrity, rendered such an operation very painful. Operation was done the next day, December 4th. The small blade of a Jones speculum was introduced ; the patient being in the Simon's posture, the urethra was by this means exposed in its entire length. A longitudinal incision was made through the mucous membrane along the dorsum of the urethra from a point where the presenting part of the mass was eroded to the base of the bladder. Another incision through the mucous membrane was made at right angles to the foregoing at a point far enough above the eroded mass to insure healthy tissue. The mucous membrane was then dissected back in two lateral flaps and the urethra was enucleated. The urethra was found to be distinctly conical in shape, the base of the cone being at the meatus, the apex at the bladder. Care was taken to dissect out the canal to a point manifestly above the zone of malignant involvement. When this point was reached, but a slight distance from the bladder, the canal, with the neoplastic walls, was excised. The cut margin of the cystic segment of the canal was seized at various points in its circumference by Kocher's forceps, brought down by gentle traction, and fixed by interrupted sutures of silkworm gut to the vaginal mucous membrane. A self-retaining catheter was inserted and the patient was put to bed. The sutures were removed on the eighth day. The catheter was dispensed with on the twelfth day. The patient sat up on the fourteenth day, when she found that she could retain her urine and void it at will. She was dismissed December 21st, entirely healed. She remained in good health until the 1st of July following—seven months—when she again summoned Dr. Morris because of some stomach symptoms. He found her suffering from persistent vomiting, and with a large mass in the epigastrium. This mass rapidly increased in size until it occupied all of the area between the navel and the breast bone, its nodular characteristics becoming more and more pronounced. She died of exhaustion, July 14, 1896, having had no recurrence whatever of the urethral trouble. No autopsy was permitted.

### SUTURE OF LARGE VESSELS INJURED IN OPERATIONS.

Dr. J. B. Murphy, of Chicago, demonstrated the method employed by him. He said in 1762 Lembert conceived the idea of suturing injuries to vessels. He made two experiments, in both of which he failed. Dr. Murphy then referred briefly to the experimental work of other surgeons along this line, pointing out their successes and failures. His own researches and operative work lead him to believe that, when a large vessel is injured in an operation necessitating a transverse division of it, not exceeding two-thirds of its circumference, the surgeon can resort to immediate suture without resection, and, if the field of operation be aseptic, can feel more certain that he will have union of the vessel and continuation of the current than he could when he sutures the intestine as for resection of the bowel. He believes from his observations that the chances are better with the suture. The importance of this concerns surgeons more in the treatment of aneurisms. Coming to the question of stab and bullet wounds of the extremities, he said there was a great field for improvement in past operative work. Formerly we ligated vessels, and when this was done the inevitable result was death of the limb. He believes that now such limbs can be uniformly saved, particularly in the aseptic cases. With his present method of suturing large vessels, he is not afraid to suture any vessel in the body, feeling confident that adhesion or union will take place.

### CONTUSIONS OF THE ABDOMEN.

Dr. W. G. Macdonald, of Albany, N. Y., presented a communication with this title. Contusions of the abdomen, he said, are always grave injuries. The question of surgical intervention, although much discussed, cannot be regarded as satisfactorily settled. Seven cases of traumatic rupture of the stomach and small intestine were reported. Two operations were undertaken, with one recovery and one death the eighth day after operation from second rupture. All the inoperative cases resulted fatally. Reference was made to the general absence of evidence of contusions in the abdominal walls when serious visceral injury has occurred. Very slight causes, particularly if the intestinal canal is distended with fluids, may produce intestinal rupture, as the falling out of bed, a blow from a barrow handle. The early symptoms of intestinal laceration are not always distinctive. An analysis of 200 cases of intestinal laceration as associated with abdominal contusion was made with a view to determining the symptoms. The following topics are considered the important ones: History of the nature of the injury, shock or collapse, pain, vomiting, pulse, temperature and physical signs. Careful investigation of a given case will usually show sufficient symptoms to make an early exploratory abdominal section imperative.

### ELECTION OF OFFICERS.

The following officers were elected: President, Dr. James F. W. Ross, Toronto, Can.; first vice-president, Dr. George Ben Johnston, Richmond, Va.; second vice-president, Dr. John C.

Sexton, Rushville, Ind.; secretary, Dr. William Warren Potter, Buffalo, N. Y.; treasurer, Dr. X. O. Werder, Pittsburg, Pa.

The next meeting will be held at Niagara Falls, on August 17, 18, 19 and 20, 1897.

